# Annotations

**Published:** 1899-10-28

**Authors:** 


### Annotations.

### Meat and Cancer.

The question how far the increase of cancer among the more highly civilised nations, which has appeared so marked during recent years, is caused by the greater quantity of meat which is eaten in prosperous countries is a matter of very considerable interest. That man is by nature not a vegetarian, in other words that he is naturally a mixed feeder, may be taken as pretty certain; but it is also certain that, as the result of hard necessity, meat has generally in times past been to him a luxury, and has but rarely formed a preponderating element in his diet. But with prosperity, and with the greater ease with which animal food can now be obtained, we find whole nations falling upon meat as a necessary ingredient of their daily diet; and in the very nations among whom this change is most noticeable we are told that cancer shows its greatest increase. In support of the connection between meat and cancer Dr. Roger Williams points to the rarity of cancer in prisons and workhouses, where but little animal food is allowed and hard work is exacted. He has lately found additional evidence as to the small amount of cancer among the convict population, which he says strongly supports this view. Taking the reports of the Commissioners of Prisons for the years 1897 and 1898, he finds that out of 5,915 convicts only 3 died from malignant disease. Thus, while the cancer mortality of convicts was 1 in 1,971, that of the general population was 1 in 698, or nearly three times as great. Before we accept these figures as exactly representing the true proportion one would like to have more details in regard to age distribution, but the difference is so striking that it is hardly likely to be materially upset by any such nicety of calculation. We need scarcely say, however, that criminals are a somewhat "selected" class of people, and that diet is not the only matter in which the convict differs from those outside the prison walls.

### The Profession and the War.

The announcement made by Mr. Wyndham on Tuesday, from his place in Parliament, that Sir William MacCormac, K.C.V.O., President of the Royal College of Surgeons, had volunteered his services to accompany the Army Corps sent out under command of Sir Redvers Buller, is one which has been received with the highest gratification by the nation at large, and especially by the medical profession. It is, indeed, a signal proof of the devotion of the medical profession to the calls of duty, of its patriotism, and of its readiness to go anywhere and do anything that may be required for the relief of suffering and for the good of humanity, that so soon as the news arrived that wounded men were lying in South Africa in want of the very highest surgical skill attainable the President of the College of Surgeons, who has but to hold up his hand to make scores of skilful surgeons follow his lead, should volunteer for the front. The circumstances are so peculiar that it is worth while to refer to them, showing as they do what poor prophets they are who leave out of their calculations those waves of patriotism and national feeling which always arise in times of war and of national emergency, and become, for a time at least, the main incentives to men's actions. For years past the Army Medical Department has been permeated throughout by a feeling that it has been unjustly treated. For twenty years past, and more, demands for this, for that, and for the other have been constantly made. Such demands, of course, could not be formulated by the officers themselves, but the matter has been strongly taken up by the profession at large, with the result that for long the Army Medical Service has been under a practical boycott at the medical schools. Thanks largely to Lord Lansdowne many of the grievances have now been removed, but not all. The army examinations have not yet shown themselves any too attractive; candidates for commissions have by no means come up in overwhelming numbers; and the Royal Army Medical Corps is still notoriously undermanned. "Wait till we have a war," some people have said, "and then we shall see;" and it has pointed out in no uncertain voice that if a great emergency were to arise the medical profession would have the Government in the hollow of their hand. And the war has come and the emergency has arisen. Not a great war, nor an overwhelming emergency, such as would compel the sinking of all differences, but a war of just such a magnitude as to stir up national sentiment, and make men feel that each should bear his part. And what has happened? Do we find the medical profession haggling for its rights? Do we find it posing as a trades union and demanding the concessions asked for? No; thank God! We find the medical profession, as represented by the president of its great college, sinking all differences and volunteering for the front. It is well done, Sir William MacCormac. We are all loyal to our profession, but our country must come first.

### Public Mortuaries.

The action of the Corporation of Glasgow in taking steps for the erection of public mortuaries will commend itself to all who have come much in contact with the poor, and have any notions of hygiene. Death in the houses of the poor is apt to lose its solemnity in mere offensiveness. To see the sheeted corpse at one end of the table, while the mother prepares the dinner, or the family eat it, at the other, is nothing uncommon. Then the length of time that a dead body is kept in the room where the living dwell, eat, and sleep, is horribly unhealthy. We know of one case where the body of a child was kept for ten days, and that in high summer. For cases such as these—and they might be multiplied by the hundred—a public mortuary would be a boon. But the question is, would the people for whom they are intended make use of them? The etiquette of death is very strict among the poor. They must show their respect for the one who is gone by keeping the shell from which the winged spirit has fled in their sight as long as possible. They must call in all their neighbours to view the corpse. They must spend as much as would keep the family in comfort for a month on useless funeral paraphernalia. And if they fail to do all this they will forfeit the respect of their social circle, which is a matter of as much importance to them as to any one else. All these considerations tend to make them object to the use of mortuaries, which are favourable to health, but offer no opportunities for emotion.

